# Conflict over non-partitioned resources may explain between-species differences in declines: the anthropogenic competition hypothesis

**DOI:** 10.1007/s00265-017-2327-z

**Published:** 2017-06-10

**Authors:** Andrew D. Higginson

**Affiliations:** Centre for Research in Animal Behaviour, College of Life and Environmental Sciences, University of Exeter, Exeter, EX4 4QG UK

**Keywords:** Anthropogenic degradation, Competitive exclusion, Environmental change, Evolutionarily stable strategy, Pollinator conservation, Resource partitioning, Seasonal breeding, Species declines

## Abstract

**Abstract:**

Human alterations of habitats are causing declines in many species worldwide. The extent of declines varies greatly among closely related species, for often unknown reasons that must be understood in order to maintain biodiversity. An overlooked factor is that seasonally breeding species compete for nest sites, which are increasingly limited in many anthropogenically degraded environments. I used evolutionary game theory to predict the outcome of competition between individuals that differ in their competitive ability and timing of nesting. A range of species following evolutionarily stable strategies can co-exist when there are sufficient nest sites, but my model predicts that a reduction in nest site availability has greater impacts on late-nesting species, especially the stronger competitors, whereas early-nesting, stronger species decline only slightly. These predictions are supported by data on 221 bird and 43 bumblebee species worldwide. Restoration and provision of nest sites should be an urgent priority in conservation efforts. More broadly, these results indicate a new ecological principle of potentially widespread importance: rapid reductions in the abundance of resources for which species’ preferences have not diversified will result in unprecedented conflicts that reduce the potential for species co-existence.

**Significance statement:**

Understanding the causes of species declines is crucial to preventing the losses. Whilst much work on species vulnerability shows broad scale effects, an enduring mystery is the variation in population trends between closely related species. I combined evolutionary modelling with three global-scale long-term data sets to reveal that competition for scarce nest sites causes variation in declines. The impact of the loss of nest sites on differential declines among closely related species from very different taxa indicates a new ecological principle of widespread importance: the effect of habitat degradation on competition among species. A lack of differentiation of nest site preferences means that—now nest sites are more limited—some species may be driving others to extinction. This phenomenon is likely to occur for any other non-partitioned resources that rapidly, on an evolutionary timescale, are now limiting population sizes.

**Electronic supplementary material:**

The online version of this article (doi:10.1007/s00265-017-2327-z) contains supplementary material, which is available to authorized users.

## Introduction

Human alterations to ecosystems are having deleterious effects on many of the world’s animal species (Butchart et al. 2010). Declines of terrestrial species have been shown to be attributable to many factors, including agricultural intensification which has reduced food abundance and diversity (Williams and Obsorne 2009; Pocock 2011), the widespread use of pesticides (Whitehorn et al. 2012; Chiron et al. 2014), the introduction of new parasites (Goulson et al. 2008a) and impacts of climatic change (Hegland et al. 2009). Previous studies have revealed that preference for different habitats may cause some species to be more vulnerable than others, but for any local fauna living in the same habitat, not all species have declined and some are even increasing (Williams 2009; Jiguet et al. 2010; Inger et al. 2014). Understanding this variability is critical not only for maintaining biodiversity *per se* but for assessing the impacts of biodiversity on other species.

The role of maladaptive behaviour has typically not been considered when trying to understand variation in species declines. Changes to environments may cause animals to make maladaptive decisions because they follow evolved rules that may be suboptimal in artificial environments (Fawcett et al. 2014), so-called ‘ecological traps’ (Robertson et al. 2013). For instance, cues indicating resource quality may become unreliable, meaning that animals make choices which are detrimental to their reproductive success (Robertson and Hutto 2006). A clear example is the choice of suboptimal nest sites in human disturbed habitats (Kolbe and Janzen 2002; Shochat et al. 2005). If rules that animals use are maladaptive in altered environments and if such rules differ among species, then following such rules could cause variation in declines. One potential cause of the variation in decision rules may be competition with other species (McNamara 2013).

Competition for resources by invasive species often causes the declines of native species (Butchart et al. 2010). There is some evidence that species are more likely to become invasive if they are related to a native, making it likely that they share an ecological niche (Duncan and Williams 2002; Park and Potter 2015). This would mean that they come into competition with particular native species, and so may differentially affect some native species more than others. However, often declining and increasing species are closely related (Williams 2009; Jiguet et al. 2010; Inger et al. 2014), suggesting invasive species probably do not contribute substantially to the variation in declines. Invasive species are typically a source of competition that is evolutionarily novel, in that it has not been experienced by the native species. Whilst less conspicuous, historically novel competition may occur between sympatric species due to changes in their environment that result in new facets of competition.

**Fig. 1:**
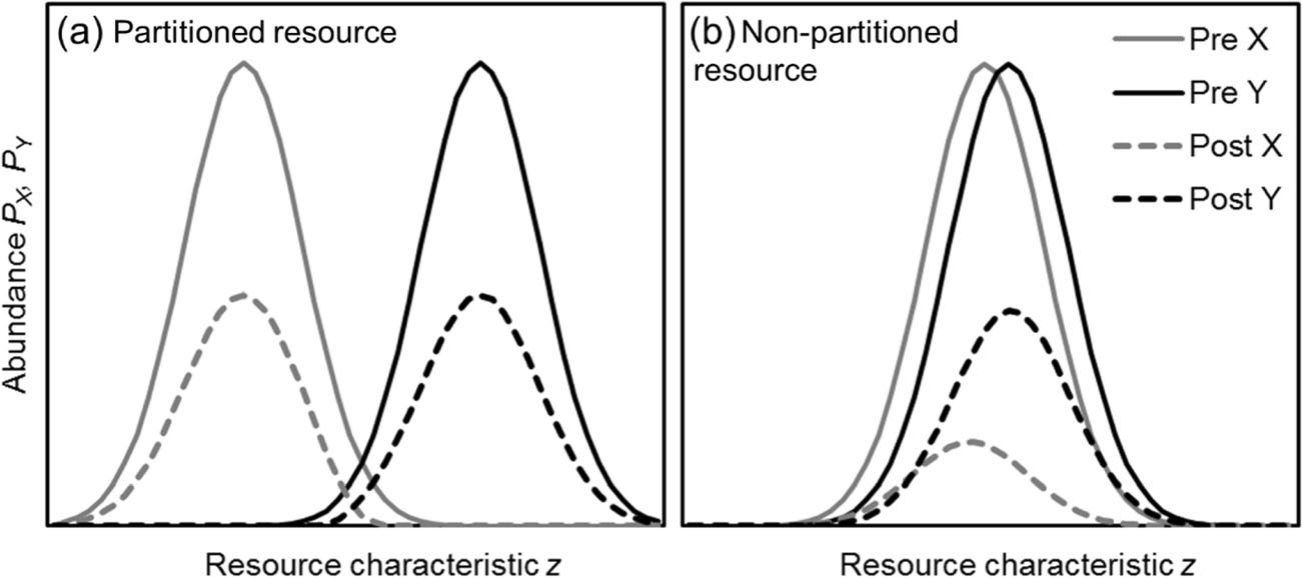
Illustration of the principle of the effect of rapid environmental change on competition between species for **a** previously limiting resources, and **b** previously non-limiting resources. Lines indicate the abundance of individuals of two species (species X: *grey*, *left*; species Y: *black*, *right*) using the resource as a function of some continuous characteristic *z*, before (*solid lines*) and after (*dashed lines*) a 50% reduction in the availability of the resource. At resource characteristic values where species overlap and so compete, a Species Y individual is assumed to be twice as likely to win in a conflict against a Species X individual. The abundance of *X* and *Y* at *z* after the decline are ${rP}_{X, z}\cdot \frac{cP_{X, z}}{cP_{X, z}+\left(1- c\right){P}_{Y, z}}$ and ${rP}_{Y, z}\cdot \frac{\left(1- c\right){P}_{Y, z}}{cP_{X, z}+\left(1- c\right){P}_{Y, z}}$, respectively, where *P*_*X,z*_ and *P*_*Y,z*_ are, respectively, the former abundance of species *X* and species *Y* at resource characteristic *z*, *r* is the availability of the resource as a proportion of former availability (*r* = 0.5) and *c* is the probability that *X* wins contests (*c* = 1/3). **a** For a partitioned resource, both species decline by around 50%. **b** For a non-partitioned resource, the competitive asymmetry causes a dramatic decline in species *X*, as shown in the present study for birds and bees competing over nest sites

Competition over important resources is a driving feature of variation in traits among sympatric species (Hutchinson 1959). Closely related species have usually evolved to avoid competition by divergent exploitation of important resources—so-called niche differentiation or resource partitioning—making co-existence possible (Hutchinson 1959). Resource partitioning may be along a single variable, such as the classic resource partitioning seen among warblers foraging at different heights in the canopy (MacArthur 1958) and variation in tongue length among bumblebee species, which has been suggested to be a cause of variation in declines (Goulson et al. 2005). Species may partition phenologically, such that their life history events (e.g. reproduction) are spread out over time. A reduction in a partitioned resource will only cause variation in the declines of related species if different types of the resource are lost more than others; otherwise, all species would decline approximately equally (Fig. 1a). Sympatric species will not have evolved partitioning for resources that are non-limiting to population sizes. So, if alteration to environments makes such resources suddenly (on an evolutionary timescale) severely limiting then sympatric species that have co-existed for many generations will suddenly be in conflict (Fig. 1b). Conflict over resources is likely to cause fast declines of less competitive species, leading to variation in population declines among closely related species.

There is evidence that bird, mammal and bee species that are less at risk have more variable or faster life histories (Gonzalez-Suarez and Revilla 2013) or start reproduction sooner in the year (Fitzpatrick et al. 2007), suggesting that differences in life history (Mayer et al. 2011) may play an important role in resilience to environmental change. Individuals of seasonally breeding species often have to search for suitable nest sites, which can limit abundance in altered environments (Newton 1994; Carvell et al. 2008). Two predominant seasonal nesting taxa that have been well studied are passerine birds and social Hymenoptera. For these taxa, nest sites are a critical resource for which individuals are highly selective (Aitken and Martin 2012). Whilst choosing an appropriate nest site when commencing reproduction is important for reproductive success (Clark and Shutler 1999; Kolbe and Janzen 2002; Lloyd 2004), among species of birds, bees and wasps that nest in similar sites (e.g. tree cavity, rodent burrow), there appears to be little resource partitioning over nest sites (Röseler and Röseler 1991; Martin and Martin 2001; Goulson et al. 2008b).

Individuals of both bird and Hymenoptera species will fight with both conspecifics and heterospecifics for access to nest sites, sometimes to the death (Richards 1978; Minot and Perrins 1986; Rӧseler and Röseler 1991; Merilä and Wiggins 1995; Carvell et al. 2008). Species show great variation in their responses to experimental manipulation of nest site availability (Aitkin and Martin 2008; Robles et al. 2012), which may occur because closely related species differ in competitive ability (Goulson 2003; Charter and Leshem 2013). Birds nest in trees, bushes and undisturbed grassland whilst bumblebees nest in long grass and abandoned burrows of small mammals. The availability of nest sites for both taxa is likely to have declined in agricultural landscapes in Europe and North America in the twentieth century due to the loss of hay meadows and rough pasture, which would remove grass tussocks and reduce rodent numbers (Harris et al. 1995), the removal of hedgerows (Pocock 2011), and harvesting of trees in mature forests (Cockle et al. 2010).

Here, I use an evolutionary model and analyses of data sets on birds and bumblebees worldwide to show that variation in decision rules determining competitive interactions—combined with a lack of resource partitioning and a decline in the availability of nest sites—can explain much of the variation among closely related species in the extent of declines. These results lead me to introduce the anthropogenic competition hypothesis: the proposition that human-induced rapid environmental changes cause variation in declines by causing sympatric species to suddenly (on an evolutionary timescale) compete over resources for which resource partitioning has not evolved.

## Methods

### The model

I constructed a model of nest establishment in which individuals of varying resource-holding potential (e.g. size) search and compete for nest sites of varying quality at different times of the season. For convenience and without loss of generality, I divide each dimension into two categories: types that are either *Big* (*B*) or *Small* (*S*) begin searching either *Early* (*E*) or *Late* (*L*) in the breeding season for nest sites that are either *Poor* (*P*) or *Good* (*G*) in terms of their associated reproductive payoff. There are more than enough nest sites for all individuals, should they find them, but individuals may die whilst searching or run out of time. I assume that Early-nesting individuals have either established a nest or died by the time Late-nesting individuals begin searching for nest sites, and that once a nest is established it cannot be taken over. In bees, the enclosure of the first worker cohort will greatly reduce the possibility of takeover (Fisher 1987). In birds, the recruitment of a mate (increasing defence) or hatching of chicks (increasing resource value to the resident) will do the same. Thus, the strategy of the Late individuals can be assessed by considering that nest site availability has declined by the number of Early individuals that have successfully established a nest. If, in the same phase of the breeding season, two individuals claim the same nest site, a fight will occur. Relative size influences the outcome of fights, with fights between a Big and a Small individual twice as likely to end in a win for the Big individual. In this context, I consider strategies that specify the decision to claim a nest site in relation to: site quality (Poor or Good), individual’s type (Big or Small, and Early or Late), size of any rival present and the current time period.

Individuals are in one of three states: floating, intruding or site-owning. Time is divided into discrete steps. Each time step the individual makes a single decision, δ, whether to search or rest (if floating) or whether or not to make a claim (if intruding or owning). There is a probability of mortality per time step spent searching (*μ*_S_) due to extrinsic factors such as adverse weather and predation (e.g. by insectivorous birds like blue and great tits *Cyanistes caeruleus* and *Parus major*), a smaller mortality rate when individuals are site-owning (*μ*_R_) and no mortality when resting. When owning or intruding, the individual can be faced by a Big, Small or no opponent and decides whether to make a claim for a nest site that it either owns or into which it has intruded.

There are differences in the ability of individuals of different species to compete for nest sites: in several social Hymenoptera, successful brood parasites tend to be larger than usurped queens, both within and between species (Goulson 2003). There may also be a resident advantage in contests (Maynard Smith and Parker 1976). I therefore assume that the probability of an individual winning a fight is:

1 $$ \gamma =\frac{1}{2}\left(1+\alpha A+\beta B\right) $$

where *α* is the effect of size on the predictability of the outcome of a fight; *A* is the difference in size between the focal individual and its opponent and takes the values −1 (opponent is bigger), 0 (opponent is same size) or +1 (opponent is smaller); *β* is the effect of residency on the predictability of the outcome of the fight and *B* takes the values −1 (focal individual is an intruder) or +1 (focal individual is a resident). I assumed *α* = 0.3, making the bigger competitor around twice as likely to win as the smaller competitor, and *β* = 0.1, giving a slight resident advantage. I assume for simplicity that the losing individual is killed, but any effect meaning that future reproductive opportunities for a defeated individual is greatly reduced (e.g. injury, exhaustion or predation) would be sufficient for the predictions to hold.

There are *T* searching opportunities in each period (Early and Late), and for the results shown I assumed *T* = 100; each could be equivalent to 1 h searching. I assume that there are more than enough nest sites for all individuals, should they find them, with equal numbers of Poor (*Q*_P_) and Good nests (*Q*_G_). The number of places to search for nest sites (*M*) determines the probability per time step (relating to nest site density) of finding a Poor and a Good nest as $\frac{Q_{\mathrm{P}}}{M}$ and $\frac{Q_{\mathrm{G}}}{M}$, respectively. I parameterize the model such that on average an individual searching in every time step would survive for 50 searches and encounter around ten suitable nest sites, five of which will be *Good*. I assume that potential reproductive success is lower in Poor nest sites than Good nest sites, due to for example space restrictions or vulnerability to predators (e.g. climbing mammals that eat eggs such as squirrels, digging carnivorous mammals such as badgers). I assume that, subject to competition with similar species, nests in Good sites produce ten individuals and those in Poor sites produce five individuals. Whilst these values may seem low for productive nests of bees, they are likely to be reasonable estimates of the mean number of new queens since most colonies fail to produce any individuals (Heinrich 2004). See Table 1 for the default values of all parameters. The absolute values of the parameters have little impact on the predictions provided the scaling is reasonable.

**Table 1:** Parameters in the model and their default values

Parameter	Symbol	Value
Maximum number of searches	*T*	100
Number of nest sites of each quality	*Q*_P_, *Q*_G_	1000, 1000
Reproductive pay off from nest type *q*	*W*_P_, *W*_G_	5, 10
Proportional decline in number of each nest type	*d*_P_, *d*_G_	0.75, 0.75
Number of places to search	*M*	10,000
Maximum individual production of each species type	*K*	500
Mortality rate when searching	*μ*_S_	0.02
Mortality rate when resident	*μ*_R_	0.001
Size bias in fight outcome	*α*	0.3
Ownership bias in fight outcome	*β*	0.1

Using standard dynamic programming techniques (see Supplementary Information), I find the evolutionarily stable strategy (ESS) for an Early-nesting individual. A strategy is an array of decisions *δ* for all combinations of the states size (Big or Small), status (*Searcher* or *Owner* or *Intruder*), opponent (Big, Small or none) and nest site (Poor or Good), for each time interval *t*. The ESS is found as follows. Starting from some arbitrary population strategy, I use forward iterated Markov chains to calculate the number of individuals in each state at each *t*. I then find the best response by a single mutant to this distribution of states, update the resident strategy in the direction of the best response and iterate this process until the strategy does not change from one iteration to the next. At this point, the strategy is the best response to itself and is therefore an ESS. I then repeat this process for Late individuals, where the only difference is that the number of available nest sites is reduced by the number already occupied by Early individuals.

To find the stable abundance of the four types (Early Big, Early Small, Late Big, Late Small), I iterate the above procedure across a number of ‘years’, assuming a finite population size. The number of individuals of each type is determined by their nesting success (number and quality of nest sites) in the previous year under the ESS, scaled by a limit on the population size of each type (i.e. each type has a carrying capacity *K* due to resource partitioning of foods). For clarity, I assume that all offspring are female; males are ignored. The abundance of the four types typically stabilizes within 10 years. To assess the change in population sizes that result from a decline in nest site abundance, I then continue running forwards for 60 years, assuming a linear decline in the number of nest sites resembling the period of increasingly intensive farming in Europe. The predictions about species trends are unchanged if the decline is assumed to be non-linear: either sudden, decelerating or accelerating. Note that I assume that individuals are not flexible; they cannot adjust their behaviour in response to nest sites becoming more scarce than they have evolved to ‘expect’. See online supplementary information for details of model implementation.

### Data collection and analysis

The novel prediction of an interaction between body size and nesting time in determining population declines was tested using published data on population trends of 147 North American and 74 European Spring-breeding small (<75 g) bird species, and proportional change in distribution of 43 bumblebee species across five countries (61 species-country combinations). All data on the explanatory variables were recorded blind to the species decline data.

#### Birds

Data on body mass, nesting period, nest type and habitat type were taken from the *Handbook of Birds of the World*, checked by personal communication from Rob Robinson at the British Trust for Ornithology. Male body masses were used as males would usually be the sex most involved in the aggressive interactions. The mean mass was used or, if the range were given instead, the average of the minimum and maximum. The earliest nesting time was taken to the first month of the range given. Each month was given the number in the year (e.g. April = 4). If the month was preceded by the word ‘early’ (or equivalent) then one third was deducted from this number. If the month was preceded by the word ‘late’ (or equivalent) then one third was added to this number. From the description of the nest, the nest type was given the categories ‘branch’ (e.g. twig cup on branch, stick platform, grass ball in reeds), ‘cavity’ (e.g. crevice between branches, cavity in tree) or ‘ground’ (e.g. depression, scrape, cup on ground) or ‘underground’ (e.g. excavation, burrow). Birds that excavate their own cavity (e.g. woodpeckers) were omitted from the analysis. Habitat type was categorized from the description or geographic location as ‘farmland’, ‘prairie’, ‘scrub’, ‘upland’, ‘wetland’ or ‘woodland’. Data on population trends were taken from the North American Breeding Bird Survey (Sauer et al. 2014) which gives trends since 1966 (only species for which the data had no deficiencies were used) as interval-specific weighted average of population change (see http://www.mbr-pwrc.usgs.gov/bbs/ for details), and from the Pan-European Common Bird Monitoring Scheme (http://www.ebcc.info/pecbm.html) which gives trends from 1980 in terms of percentage change.

Analysis of the North American fauna and the European fauna were carried out separately. The distribution of body mass suggested a natural gap above 75 g, so to ensure competition could occur, I only considered species of 75 g or lighter. In order to provide an appropriate test of the model, only species that start nesting in Spring were used (late February to late May). Data on 74 European species and 147 North American species met all the criteria. I constructed nested general linear mixed models using R packages lme4 and lmerTest (Bates et al. 2015; Kuznetsova et al. 2015), including the various effects and interactions and compared the model fit following standard techniques. In both cases, including nest site preference and habitat type in the models significantly improved the fit. As I was not interested in the effect of habitat and the species with data led to an arbitrary number per habitat, I entered habitat as a random factor. Nest type was predicted to affect the declines because different nest sites will have changed by differing amounts, so nest type was entered as a fixed factor. Given that I had a priori prediction about the direction of the interaction, *P* values for the interaction and the main effects of body size and nesting time were adjusted following the directed test approach (Rice and Gaines 1994): i.e. the two-tailed *P* value was multiplied by 0.625.

#### Bumblebees

Relative declines in bumblebee species were taken from the literature (Fitzpatrick et al. 2007; Williams 2009; Cameron et al. 2011) for five geographic locations: Britain, Canada, China, Ireland and the USA. Note that not all time periods considered are the same; see the original papers for details. To allow comparison between data sets, I converted all data to proportional change in incidence across the study area, relative to the pre-1960 distribution. Data on individual body length, individual tongue length, month of individual’s emergence from winter torpor and species nest site preference were taken from the literature (Goulson et al. 2005; Williams 2009; Colla et al. 2011; Koch 2011). Due to a smaller number of species, and species occurring in several data sets, it was impossible to carry out the analysis in the same way as for birds. Instead, values *relative* to species in the same country were used. The relative timing of emergence was calculated for each species by assigning a value of zero to the species with the median emergence time in that location; earlier species were given negative values and later species positive values. The relative size of individuals was calculated in a similar way, with the median-sized species in each location given a value of zero, smaller species given negative values and larger species positive values. These relative values are important because the model predicts that size and emergence time relative to nest site competitors should determine declines. Tongue length was categorized as ‘short’, ‘medium’ or ‘long’. Nesting preference was categorized as underground, surface or either (i.e. species that nest both above and below ground). Nesting preferences are not published for the China fauna.

Many species were recorded in more than one location and location had an effect on proportional change, so the data were analysed using general linear mixed model, with location and species as random factors. Proportional declines were normalized using an arcsine square-root transformation for all analyses. I constructed several candidate models starting with all the factors and their interactions, and compared AIC scores to find the best-supported model. The resulting best-fit model from AIC scores was the same as the minimal adequate model found from a complementary backwards stepwise process, eliminating non-significant (i.e. *P* > 0.05) interactions and factors one at a time. Thus, the models reported in the text are the best fit from both procedures.

## Results

### Model predictions

**Fig. 2:**
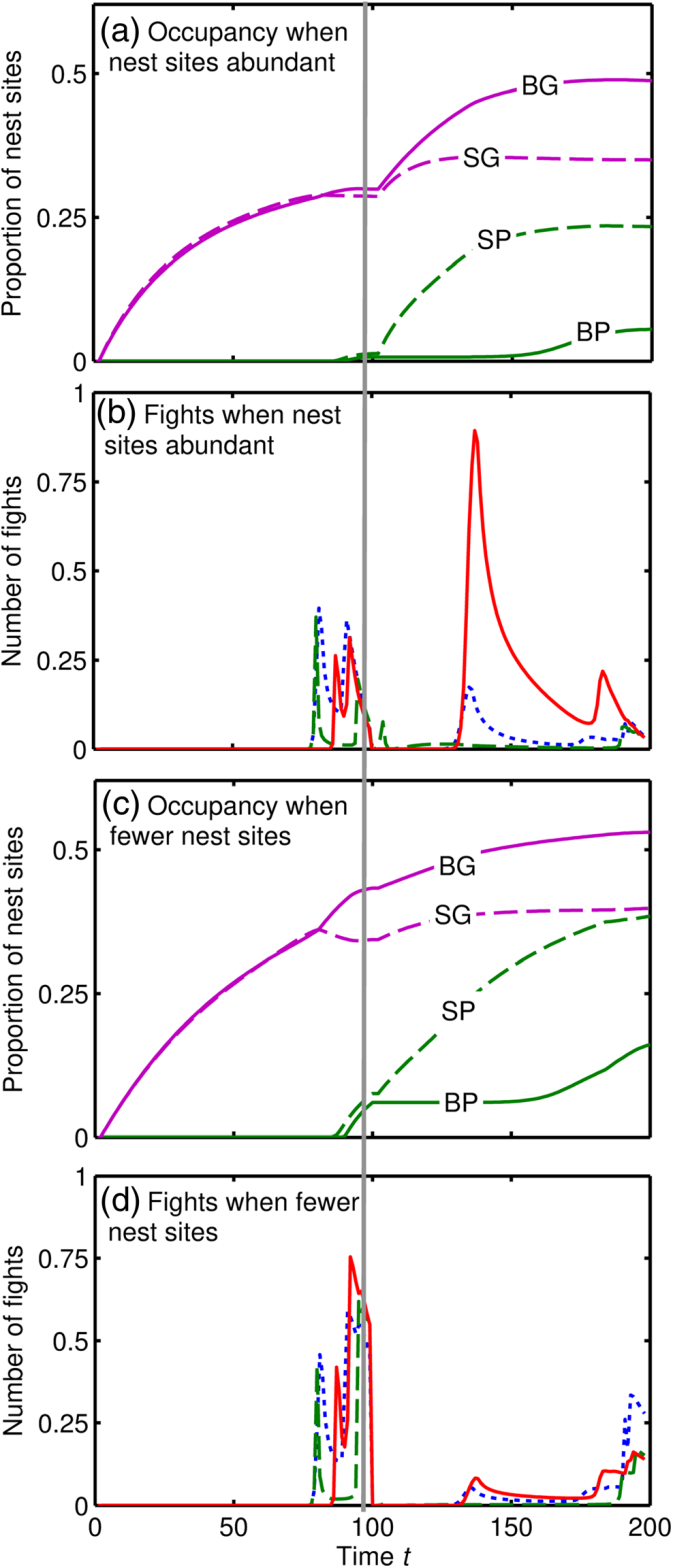
**a**, **c** Nest occupation by *Big* (*B*, *solid lines*) and *Small* (*S*, *dashed lines*) types as a proportion of the *Good* (*G*, *purple lines*) and *Poor* (*P*, *green lines*) sites over the season and **b**, **d** amount of fighting over the season showing *Small-Small* fights (*dotted blue lines*), *Big-Big* fights (*solid red lines*) and *Small-Big* fights (*dash green lines*) at **a**, **b** natural (pre-intensive agricultural) and **c**, **d** modern nest site densities. Time is shown as continuous between the *Early* (0 ≤ *t* ≤ 100) and *Late* (100 ≤ *t* ≤ 200) periods

The evolutionarily stable strategy (ESS) is shown in Table 2 for decisions in the middle of the nesting period. Generally, individuals should claim Good nest sites if they are unoccupied or the current resident is smaller than them, but avoid fighting bigger individuals. Poor sites are very little used by Early types (Fig. 2a) because individuals of neither size claim them (Table 2), and because mortality during searching reduces the number of individuals that might occupy sites. Due to the availability of Good sites, there is very little fighting between Big individuals in the Early period, whilst Small individuals fight one another for good sites near the end (Fig. 2c). As *Good* sites become occupied, most Late Small species end up occupying Poor sites, whilst Big types occupy Poor sites towards the end of the period or fight one another for the remaining Good sites. Fights between Small and Big individuals are very rare, because it is rarely worth the risk for Small individuals as the relative pay off from poor sites (50% of the payoff from good sites) is greater than the probability of winning (33%). The number of fights is very small, with a total fewer than 50 in both periods from a total population of around 1600 individuals (i.e. over 90% of individuals never fight). At the end of the Late period, a quarter of Good sites and three-quarters of Poor sites remain empty.

**Table 2:** Evolutionarily stable decisions about whether to claim a Poor or Good nest site in the middle of the Early or Late-nesting period for Small and Big individuals when there is no opponent (N), a small opponent (S) or a big opponent (B). The decision is denoted as: always claim (A), never claim (N) or claim only if current resident (R)

			Opponent		
Nesting period	Nest quality	Focal	None	S	B
Early	Poor	Small	N	N	N
Early	Poor	Big	N	N	N
Early	Good	Small	A	R	N
Early	Good	Big	A	A	R
Late	Poor	Small	A	R	N
Late	Poor	Big	N	N	N
Late	Good	Small	A	R	N
Late	Good	Big	A	A	A

**Fig. 3:**
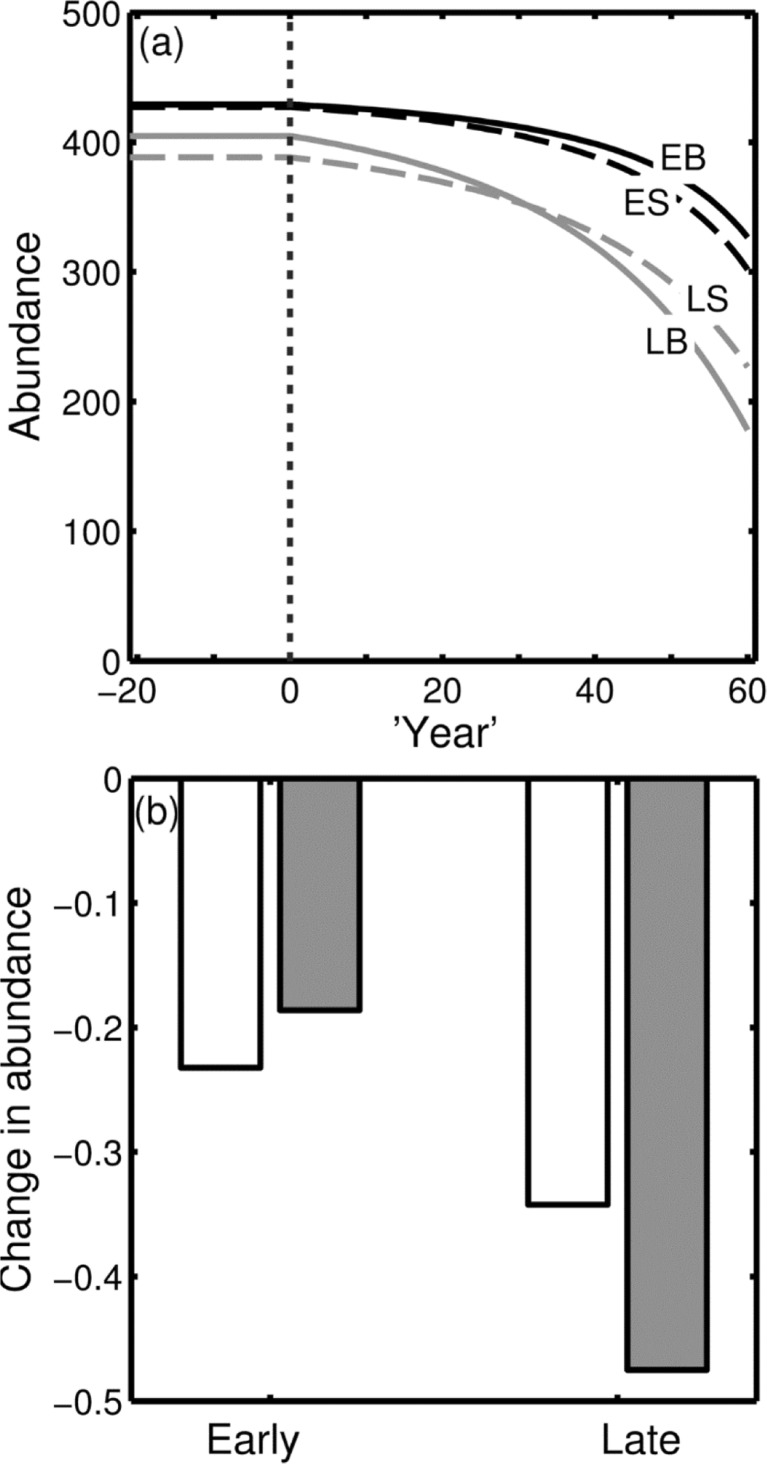
**a** Trajectory of abundance over the simulated time period showing the stable abundance of each type under natural nest site density and the effect of gradually declining nest site density over the following 60 years. Species are categorized as *Early* (*E*, *black lines*) or *Late* (*L*, *grey lines*) and *Small* (*S*, *dashed lines*) or *Big* (*B*, *solid lines*). **b** Proportional change in population size over 60 years in *Big* (*grey bars*) and *Small* (*white bars*) species that emerge *Early* or *Late* (shown on the *x*-axis). All other variables take the default values shown in Table 1. The model predicts that late-nesting species will decline more than early-nesting species, but that this effect will be far greater for big species than small species

The ESS leads to stable abundances of the four types (Fig. 3a, to the left of the vertical dotted line); abundance is slightly greater for species with *Early* and *Big* individuals but within-type competition over food allows co-existence of types. I use the model to predict how a 75% decline in the availability of nest sites - gradually and linearly over 60 years - affects the population sizes of different species, depending on their body size and emergence time, assuming that the decision rules are unchanged.

The reduction in nest site availability causes greater use of Poor sites by both sizes, as well as proportionally greater use of Good sites by Big individuals (cf. Fig. 2a, b). Fights are slightly more common at the end of the Early period, whereas in the Late period there is a counter-intuitive reduction in fighting due to nest sites being more difficult to find (Fig. 2d). A reduction in the availability of nest sites causes a decrease in the predicted abundance of all types, which occurs at an accelerating rate due to escalating effects of competition (Fig. 3a, to the right of the vertical dotted line). At the predicted abundances after 60 years (Fig. 3b), Late species have declined the most, because they struggle to find suitable nest sites towards the end of the season. However, the model predicts a crossover interaction between the effects of body size and emergence time (Fig. 3b). The greatest decline in abundance is observed for Big Late individuals, because Big individuals are more likely to reject Poor nest sites (Table 2), but late in the season they often fail to find a Good site and so end up with zero reproductive success. In contrast, Big Early species decline little because they are able to take ownership of the limited Good sites. A sensitivity analysis (Supplementary Information) shows that this crossover interaction is expected to occur for a wide range of ecological circumstances.

To make the model tractable, I assume that individuals are unable to remember the location of nest sites that they have rejected whilst searching. If they were able to remember poor nest sites, and return to them when time becomes short, then the effects that occur in nature may be weaker than those predicted by the model. However, it is possible that I have underestimated the decline in nest site density (75%) since the abundance of food sources has decline by 97% (Goulson 2003); it is likely that other resources have declined as much. Any further decline would increase the strength of the interaction (Fig. 3b). I have also assumed that individuals are only of two sizes, when in fact there is continuous variation in individual size both between and within species. However, I found that the magnitude of the effect of body size, and the effect of resident, are relatively unimportant. This occurs because there is a constant probability of finding another nest site that is unoccupied, and a chance of having no intruders for the rest of the period, so it is usually in the interest of the weaker individual to avoid fighting. Thus, absolute differences between species are likely to be unimportant in driving differential declines.

### Tests of model predictions

#### North American birds

**Fig. 4:**
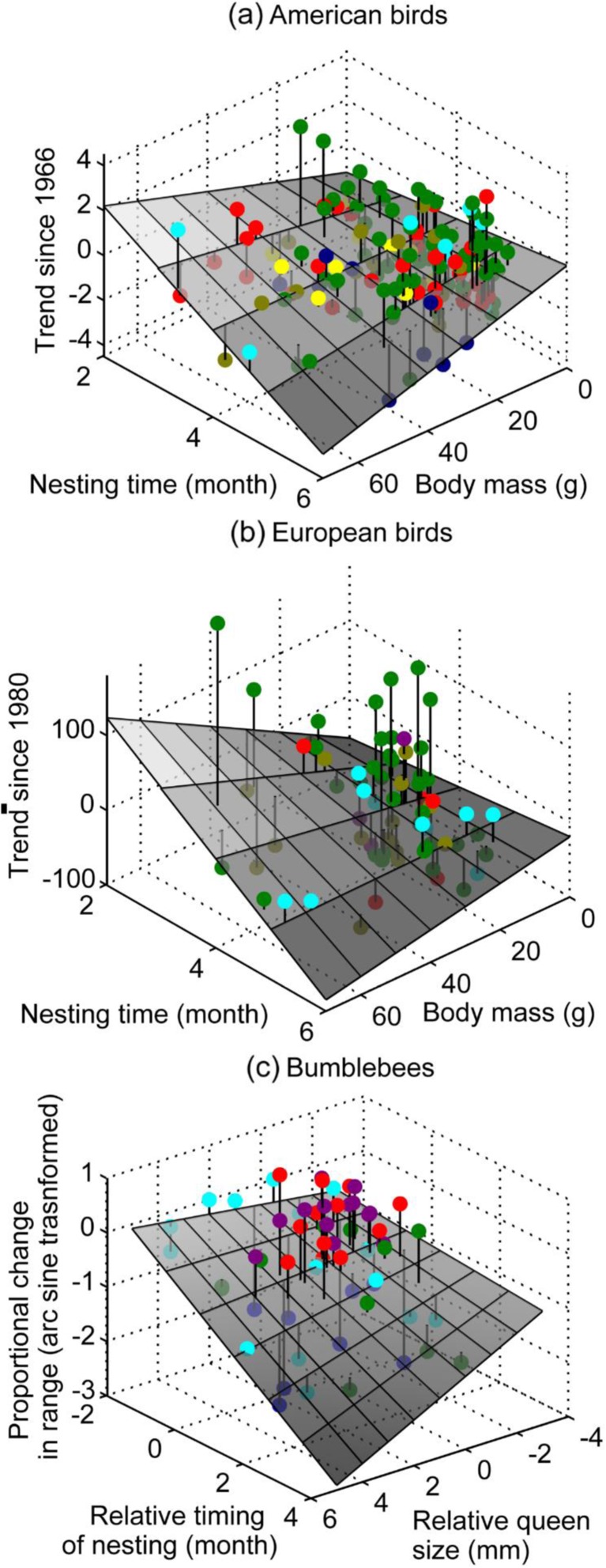
Population trends of **a** 147 North American birds (weighted average of population change, from North American Breeding Bird Survey), **b** 74 European birds (percentage change, from the Pan-European Common Bird Monitoring Scheme) and **c** 61 bumblebees (2 × arcsine square-root transformed) plotted against (relative) nesting time and (relative) body size, with each data point representing a species in a specific habitat or location. *Shaded planes* represent the fitted regression models predicting the **a**, **b** population trends and **c** proportional change in abundance (**a**: *y* = −1.886 + 0.323 × time + 0.098 × mass − 0.025 × mass × time; **b**: *y* = −75.208 + 9.281 × time + 4.311 × mass − 0.874 × mass × time; **c**: *y* = −0.818 − 0.211 × relative size − 0.042 × relative time − 0.052 × relative size × relative time; note that nest site type is omitted from these models); *vertical lines* indicate the error in predictions. *Coloured dots* indicate **a** habitat: desert = *yellow*; farmland = *olive brown*; prairie = *navy blue*; scrub = *red*; wetland = *cyan*, woodland = *green*; **b** habitat: farmland = *olive brown*; scrub = *red*; upland = *purple*, wetland = *cyan*, woodland = *green*; **c** countries: USA = *blue*, Britain = *cyan*, Canada = *red*, China = *purple*, Ireland = *green*

The species population trend since 1960 among small birds was different among habitat types (*F*_5,144_ = 5.245, *P* < 0.001) because prairie and farmland birds have declined more than wetland and woodland birds. There were differences among nest types (*F*_2,141_ = 3.090, *P* = 0.049) with ground-nesting birds having declined more than branch- or cavity-nesting birds, but there was no interaction between nest type and habitat (*F*_9,139_ = 1.600, *P* = 0.122). Due to the impact of habitat and because I was specifically expecting an interaction with nest type, I fitted a linear model with nest type as a fixed factor and habitat type as a random factor. This showed that the population trend becomes more positive the heavier the species (*t*_132.4_ = 3.430, *P* = 0.001), indicating that small species have declined most. There was a borderline main effect of nesting time (*t*_131.9_ = 1.788, *P* = 0.048), and the interaction between nesting time and body mass was significant (*t*_132.1_ = −3.217, *P* = 0.001), indicating that the effect of body mass is reversed among later-nesting species (Fig. 4a). There was also a three-way interaction between nest type, nesting time and body mass (*t*_131_ = 2.596, *P* = 0.010). Separate analyses showed that this occurred because the interaction was highly significant for cavity-nesting species (*t*_34_ = −3.659, *P* < 0.001), significant for ground-nesting species (*t*_22_ = −2.376, *P* = 0.016) and non-significant for branch nesting species (*t*_75_ = −0.838, *P* = 0.253). For full details, see Table S1.

#### European birds

The species population trend since 1980 among European small birds was not significantly different among habitat types (*F*_3,70_ = 1.629, *P* = 0.191), but upland and farmland birds have tended to decline more than wetland and woodland birds. There was a suggestion of a difference among nest types (*F*_2,69_ = 2.635, *P* = 0.079), again because of ground-nesting birds having declined more than branch- or cavity-nesting birds, but there was no interaction between nest type and habitat (*F*_6,67_ = 0.948, *P* = 0.468). A model with nest type as a fixed factor (including interactions) and habitat type as a random factor revealed that the trend becomes more positive the heavier the species (*t*_60.4_ = 2.695, *P* = 0.006), indicating that small species again have declined most. Again, there was evidence for the predicted interaction (*t*_60.7_ = −2.535, *P* = 0.008), indicating that the effect of mass is reversed for later-nesting species (Fig. 4b). There was no main effect of nesting time (*t*_61_ = 1.600, *P* = 0.072). Again there was a three-way interaction between nest type, nesting time and body mass (*t*_61_ = 2.561, *P* = 0.022). Separate analyses showed that this occurred because the interaction was almost significant for cavity-nesting species (*t*_19.3_ = −1.933, *P* = 0.088), significant for branch nesting species (*t*_23.7_ = −2.632, *P* = 0.009) and non-significant for ground-nesting species (*t*_14.3_ = 0.519, *P* = 0.421). For full details, see Table S2.

#### Worldwide bumblebees

Data on bumblebee species were from five countries with overlapping fauna, so timing of nesting and body size were assigned values relative to sympatric species, to make the data comparable. The overall mean proportional change in bumblebee abundance is −0.216, with great variation among species: some species are almost locally extinct (most negative −0.985 for *Bombus occidentalis* in the USA and −0.733 for *Bombus subterraneous* in Britain) whereas others have increased their distribution (most positive +0.101 for *Bombus impatiens* in Canada and +0.105 for *Bombus friseanus* in China). The proportional change in abundance becomes more negative the later the relative emergence time (*t*_47_ = −3.818, *P* < 0.001). There was no main effect of relative body size (*t*_41.6_ = −1.15, *P* = 0.160), but there was an interaction between relative time and relative size (*β =* −0.052, *t*_52.4_ = −1.970, *P* = 0.034). Among early-nesting species, the proportional change becomes more positive (representing a bigger increase) as relative size increases. Conversely, among late-nesting species, the proportional change becomes more negative as relative size increases (Fig. 4c). There was no effect of location on this interaction (*χ* ^2^_12_ = 15.376, *P* = 0.221).

Bumblebee species that nest strictly underground have declined more than species that are strictly or facultatively surface-nesting (omitting the Chinese fauna: *t*_35.7_ = 3.046-, *P* = 0.004). The interaction between relative size and relative time was stronger if nest type was included in the model (assigning the Chinese fauna, for which nesting preference is not known, as all underground-nesting: *β =* −0.055, *P* = 0.018, or all strictly or facultatively surface-nesting: *β =* −0.055, *P* = 0.018, or randomly to underground or surface-nesting with probability based on the proportions in other countries (22/48 = 0.541 to underground): *β =* −0.054, *P* = 0.023). For full details, see Table S3.

No significant effects of size (*t*_51_ = −1.135, *P* = 0.262), time (*t*_55_ = −0.609, *P* = 0.545) or their interaction (*t*_55.4_ = −0.217, *P* = 0.829) were found when using raw individual body length and month of emergence instead of their relative values. This suggests that it is something about competition, rather than specific requirements (e.g. diet), that determines declines. Contrary to previous suggestions that variation in declines relates to variation in flower handling (Williams 1986; Goulson et al. 2005; Kleijn and Raemakers 2008), tongue length had no significant effect on the pattern of declines (*χ* ^2^_2_ = 1.144, *P* = 0.564). However, tongue length is related to relative size (*F*_1,2_ = 4.249, *P* = 0.019), so in theory this could be driving the observed effects. To check this, I replaced relative size with tongue length, and vice versa, in the maximal model and the minimum adequate model. In both cases, using tongue length resulted in poorer fit (maximal: ΔAIC = +3.1; minimum adequate: ΔAIC = +4.4). In addition, there was no main effect of tongue length (*F*_2,57_ = 1.674, *P* = 0.182), nor interactions with relative time (*χ* ^2^_2_ = 3.644, *P* = 0.162).

In summary, all three data sets support the model’s novel prediction that population declines are influenced by a crossover interaction between body size and nesting time.

## Discussion

Rapid environmental change provides challenges to which species may be unable to adapt due to limitations on plasticity in behaviour (Sih et al. 2011). Up to now, most studies have focused on the responses of individuals to their environment (Sih et al. 2011). For instance, species may choose maladaptive locations to settle, known as ecological traps (Robertson and Hutto 2006). The study of invasive species highlights the importance of competition with other animals (Murray et al. 2014). But competitive exclusion could occur not only because one of more species is novel, but because the competed resource is novel, in that animals do not have decision strategies that take the resource limitation into account because they are not adapted to altered environments (Fawcett et al. 2014). Here, I have provided the first evidence that this phenomenon—anthropogenic competition—is important at determining species vulnerability.

Whilst threats to species from environmental change are often overlooked (Murray et al. 2014), my findings imply that such change is likely to interact in complex ways with interspecific behavioural interactions to determine species vulnerability. The impact of the loss of nest sites on differential declines among closely related species from very different taxa (birds and bees) illustrates a principle of potentially widespread importance: the effect of rapid anthropogenic environmental change on competition among species (Fig. 1). Competition between species over evolutionary time should lead to resource partitioning. If so, a reduction in resource availability will tend to reduce all species by an equal proportion (Fig. 1a). In the case of nest sites of birds and bees that nest in similar sites (e.g. tree cavity, rodent hole), there appears to be no—or at most partial—resource partitioning (Richards 1978; Nilsson 1984; Munro and Rounds 1985), which implies that nest sites have not been severely limiting over evolutionary time, and species differences in behaviour have been sufficient to prevent resource partitioning. Hence, now that nest sites are severely limited and animals are more likely to compete for them, species that happen to be larger or more aggressive may drive others to extinction (Fig. 1b). This anthropogenic competition is likely to occur for any other non-partitioned resources that suddenly—on an evolutionary timescale—are now limiting population sizes, and so may be an important and ubiquitous driver of differences in the vulnerability of the world’s animals and plant species to environmental change.

### Evaluating the model

Seasonally nesting species experience strong selection to search for high-quality nest sites (Newton 1994; Martin and Martin 2001; Kolbe and Janzen 2002; Both and Visser 2003). Rapid degradation of the environment over recent decades, to which it is particularly challenging for animals to adapt (Sih et al. 2011), may have led to nest site selection strategies becoming maladaptive. This assumption was made for two reasons. Firstly, if nest sites were always abundant in evolutionary history, such that there was never a serious risk of failing to find a high-quality one given sufficient searching, then animals are unlikely to have a strategy that responds to availability in any given year. Secondly, progress has been made in understanding species’ responses to environmental change by the assumption that behaviour is not completely plastic (so-called human-induced rapid environment change (HIREC) (Sih et al. 2011)), so this is a common assumption in many useful studies. Note that I do not assume that any animal has an optimal strategy as codified in the model, but that natural selection will have given them decision rules that approximate this strategy (Fawcett et al. 2014). Selection on this rule may alter species’ behaviour, and so may aid in the apparent recovery of species.

However, it is possible that nest selection strategies could still be adaptive under changed environment conditions (e.g. species learn), but performance is still poorer for some species than others. Or it could be that strategies have altered in response to changed environmental conditions, but performance is still poorer. Future developments should attempt to assess whether these two possibilities make different predictions in terms of behaviour or population trends. There are at least two reasons to expect that including such flexibility would not substantially alter the results. Firstly, if animals do respond to nest site availability, then changes in choosiness may be similar for all species, and hence minimally affect the qualitative predictions. Secondly, learning that nest site availability is reduced will take time, meaning that the animal must search for a long time before altering its behaviour. Under the current assumptions, merely the fact that they are still searching indicates that nest site availability is low, so the strategy already takes reducing availability into account and is likely to not substantially change.

Competition over nest sites may seem unlikely to exert strong selection without a significant amount of mortality due to fighting, but the model shows that even a low incidence of fighting may generate an important selective pressure, analogous to the strong indirect effect of predation risk on behaviour and life history in many species (Preisser et al. 2005). The declines are driven not by mortality during fights, which are rare, but by mortality whilst searching for nest sites and failure to find an acceptable nest site before it is too late. Large, late-nesting species have declined the most because they ‘rely’ on their superior fighting ability to take over high-quality nests late in the season. In the natural environment (i.e., prior to agricultural intensification), this strategy was adaptive, but with the reduced nest site availability in modern environments such species may leave it too late to fight, or accept a poor nest site and/or die searching. Moreover, the hypothesis does not imply saturation of nest sites, as has been asserted to be necessary for a role of nest sites in limiting populations of both birds and bees (Walankiewicz 1991; Goulson 2003). Because they have limited knowledge of nest sites and limited time, individuals will be randomly clumped (e.g. the numbers of individuals finding each nest site will follow a Poisson distribution) rather than evenly distributed across nest sites, leading to more competition than would occur if individuals were omniscient.

### Evidence for the role of competition over nest sites in species declines

The hypothesis predicts that the effect would be most significant when nest sites are harder to find, a prediction that is supported by the finding that the interaction is clearest for bird species that nest in cavities or crevices. This pattern is all the more striking because ground-nesting species have actually declined more than others, indicating that the causes of variation among species may not be the predominant cause of declines overall (general loss of habitat, (Butchart et al. 2010)). The finding that bumblebee species that nest strictly underground have declined more than others further emphasizes the importance of nest sites, since underground nest sites are likely to be more limited than surface nest sites as they depend on rodent activity, rather than only the growth of plants. Species that have increased in abundance, such as the great tit (*Parus major*) and the tree bumblebee (*Bombus hypnorum*), both commonly nest in artificial bird boxes in suburban gardens. Competition over nest sites may also be influencing the spread of invasive species if such species are large and establish nests early in the season. It is notable that three passerine species undergoing very substantial range expansion in the New World, the house sparrow *Passer domesticus* (Marzal et al. 2011), the common starling *Sturnus vulgaris* (Chamberlain et al. 2009) and the western bluebird *Sialia mexicana* (Duckworth and Badyaev 2007), are all relatively large (greater than 64, 100 and 70% of my North American species data set, respectively), aggressive, nest early in the year in cavities and are known to compete with declining species for nest sites (Weitzel 1988; Ingold 1998).

Invasive bumblebees include *Bombus terrestris* in Japan (Inoue et al. 2008) and *Bombus ruderatus* in South America (Madjidian et al. 2008), which have both been associated with declines in the abundance of native species. This is thought to be due to competition with native species over floral resources (Walankiewicz 1991; Madjidian et al. 2008), although it is unclear why they would be sufficiently competitive to cause the dramatic declines that are observed (Inoue et al. 2008, 2010). In some locations, introduced parasites seem to be important (Arbetman et al. 2013), but again it is unclear why some species would be more vulnerable than others. Both *B. terrestris* and *B. ruderatus* are large-bodied species that emerge early in the year, a combination of traits that will cause them to be highly competitive for nest sites. Furthermore, there is strong evidence that nest site availability limits population sizes (Inoue et al. 2008), providing the potential for competition over nest sites to lead to the declines of native species. It is therefore unfortunate that *B. terrestris* is the most commonly commercially bred bumblebee species (Ings et al. 2005). Two bumblebee species that have suffered huge declines in Europe are *Bombus soroeensis* and *Bombus ruderarius* (Goulson et al. 2005). Both are in the middle of their climatic range in Europe, which is inconsistent with the suggestion that species will decline if at the edge of their climatic range (Williams 2009). Both species are small and late-emerging, so may struggle to find and win increasingly scarce nest sites.

### Alternative explanations

Body size is a predictor of declines in birds but the observed pattern is inconsistent (Siriwardena et al. 1998; Pocock 2011); perhaps because the effect of size depends on the timing of nesting (Fig. 4). An important source of inconsistency in results is that body size does not perfectly correlate with competitive ability (Duckworth and Badyaev 2007) because smaller species may be more aggressive or well-armed. Pesticide use may affect different species to varying extents, with small late-emerging species thought to suffer worse (Cameron et al. 2011), which would make the observed crossover interaction *less* likely. The present study also suggests that the apparent effect of tongue length (indicating different flower preferences) on bumblebee declines (Goulson et al. 2005) may be an artefact of differences in overall body size affecting competition for nest sites, which may explain why tongue length was not a significant predictor of declines in a previous meta-analysis (Williams 2009).

An explanation for between-species differences in declines of both birds (Pocock 2011) and bees (Kleijn and Raemakers 2008) is the limited flexibility of their foraging, but the evidence is inconclusive (Williams 2005) and patterns are weak (Pocock 2011), and it would be surprising if species with flexible foraging behaviour (Goulson 2003) would be unable to change their behaviour to exploit alternative food resources. Whilst being a farmland specialist seems to make bird species more vulnerable, there is great variation among them (Siriwardena et al. 1998) that is not explained by the other factors suggested to explain bird declines, such as clutch size, length of the dependent period and diet (Siriwardena et al. 1998). Further noise results from differences in behavioural flexibility, with less flexible species declining more (Duckworth 2014). Other factors suggested to explain variable declines among bee species include climatic range, floral specialization and tongue length (Williams 1986; Goulson et al. 2005; Kleijn and Raemakers 2008), but each explanation has only mixed support (Goulson 2003; Mayer et al. 2011). In conclusion, for both birds and bees, it is potentially possible to identify a suite of factors that can combine to explain much of the variation, but the model presented here can also explain much of the variation, but with greater parsimony.

### Implications for pollinator conservation

If resource partitioning over floral resources combined with the loss of a subset of flower species is not the primary cause of variation in bee declines, our current understanding of the causal relationship between plant and pollinator declines should perhaps be reconsidered. Diversity in pollinator communities is beneficial for plant populations (Fontaine et al. 2006), so some flower species may have declined because competition for nest sites has caused declines in those pollinator species that prefer them. If this is the case, increasing nest site abundance should have resultant benefits for plant populations. The decline of pollinator populations worldwide is a major environmental and economic concern due to potential impacts on food production, environmental health and the extinction of dependent species (Burkle et al. 2013). The accelerating rate of declines in abundance predicted by the model suggests that an essential part of habitat restoration involves the creation of nest sites, suggesting that policy-makers and practitioners should include the creation of nest sites in the portfolio of conservation efforts.

## Compliance with ethical standards

ESM 1 (DOC 185 kb)
ESM 2 (DOC 139 kb)
ESM 3 (DOC 99 kb)
ESM 4 (DOC 144 kb)
ESM 5 (CSV 2 kb)
ESM 6 (CSV 2 kb)
ESM 7 (CSV 4 kb)
