# Supplementary material for: Conflict over non-partitioned resources may explain between-species differences in declines: the anthropogenic competition hypothesis
Source: Behav Ecol Sociobiol. 2017 Jun 10;71(7):99. doi: 10.1007/s00265-017-2327-z (PMC5486810; doi:10.1007/s00265-017-2327-z)
Supplement: ESM 1 — (DOC 185 kb) [file 265_2017_2327_MOESM1_ESM.doc]

**SUPPLEMENTARY INFORMATION: ANALYSES**

for

Conflict Over Non-partitioned Resources May Explain

Between-Species Differences in Declines:

The Anthropogenic Competition Hypothesis

in

Behavioral Ecology and Sociobiology

by

**Andrew D. Higginson**

Centre for Research in Animal Behaviour, College of Life and Environmental Sciences,

University of Exeter a.higginson@exeter.ac.uk

**Sensitivity Analysis**

Here, I explore the dependence of the cross-over interaction predicted in Fig. 2B on the assumed values of the critical parameters. The results of this sensitivity analysis are shown in Fig. S1. The size advantage in contests *α* has no impact on the relative declines of the four species types (Fig. S2A), whilst the resident advantage *β* has no effect unless it is much larger than *α* (Fig. S2B, and true for all *α*) because it no longer pays *Late Big* species to hold out for the best sites. The relative declines of the four types is influenced by the payoff from *Poor* sites (Fig. S2C), with *Late Big* types declining more than others only if the payoff is very different to the payoff from *Good* sites (unless the payoff is zero: i.e. there are no *Poor* sites). When all sites give similar payoffs *Big* types tend to decline less than *Small* types because *Late Big* individuals claim all nest types and so competitive ability becomes more important. The number of places to search *M,* which controls the probability of finding a nest site per time step, has no effect on the relative declines (Fig. S2D), although as *M* increases and sites become more difficult to find the effects of size and timing get smaller. The proportion of sites that have been lost that were *Good* has stronger effects on the particular pattern that we observe (Fig. S2E). When *Poor* nest sites have been lost much more than *Good* sites the decline of *Late Small* types is predicted to be greater than the decline of *Late Big* types because *Late Small* types preferentially nest in *Poor* sites. If, on the other hand, most nest-site loss is of *Good* sites, *Early* types are predicted to decline more because they preferentially occupy *Good* sites. The interaction is not found when mortality whilst searching *μ_S_* is very high (Fig. S2F) because individuals should accept the first nest they find, nor if mortality is zero because *Late Big* individuals do not suffer increased mortality when searching for scarcer *Good* sites. Thus, the model predicts the cross-over interaction for almost all reasonable parameter settings.


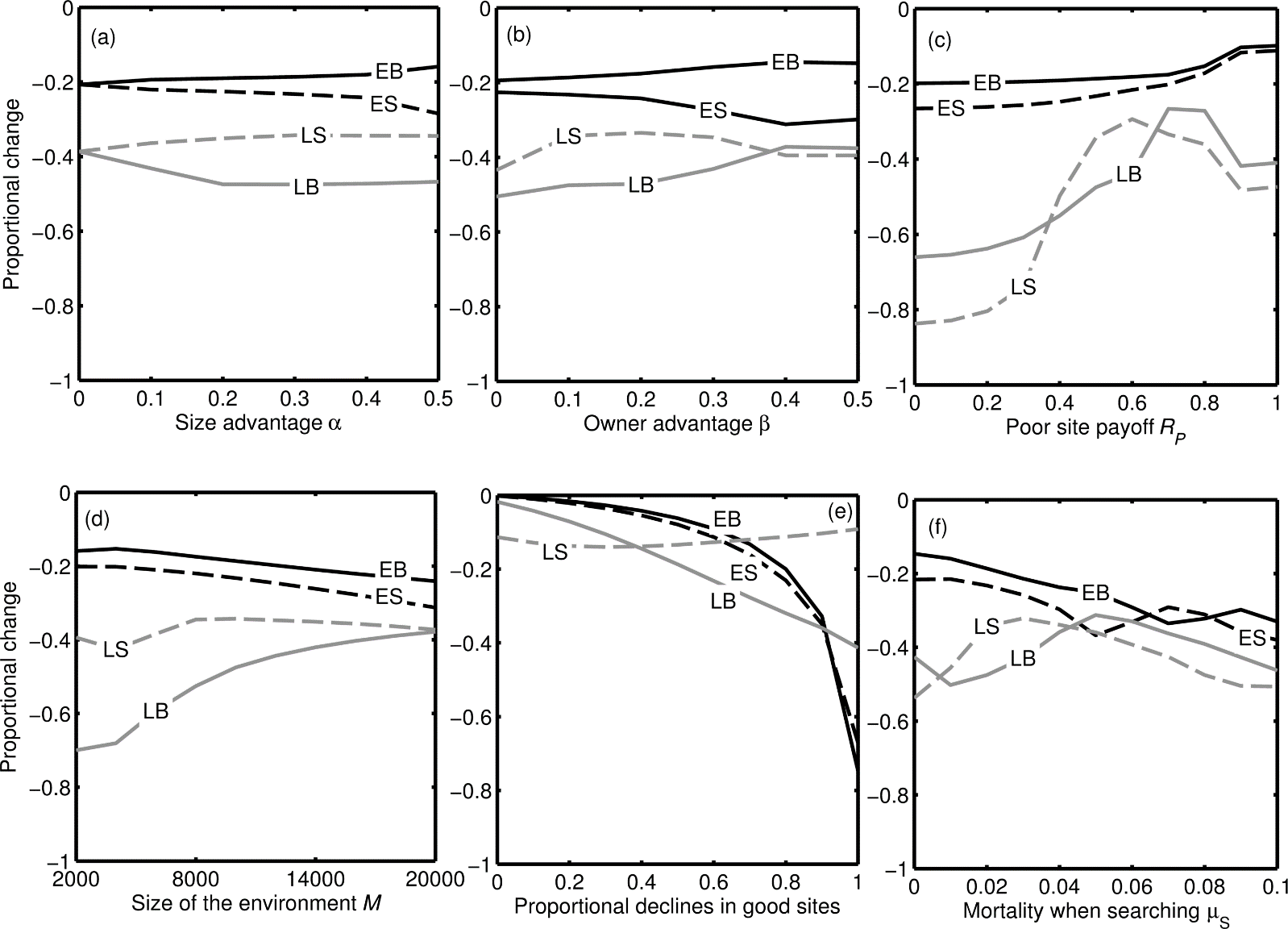


**Fig. S1:** Proportional change for the four types indicated by *Early* (*E*, black lines) or *Late* (*L*, grey lines) and *Small* (*S*, dashed lines) or *Big* (*B*, solid lines) as a function of (a) size advantage in fights *α*, (b) resident advantage in fights *β*, (c) payoff from poor nest sites *W_P_*, (d) number of places to search *M*, (e) proportional decline in the abundance of *Good* nest sites [*d_G_*/(*d_P_*+*d_G_*)], where *d_P_* + *d_G_* = 0.75, and (f) mortality rate when searching μ_S_.

Table S1: The results of fitted models for North American birds

| **Variable** | **Full model (*n*=147)** | | | | **Branch (*n*=79)** | | **Cavity (*n*=38)** | | **Ground (*n*=30)** | |
| --- | --- | --- | --- | --- | --- | --- | --- | --- | --- | --- |
|  | *β* | *t* | *d.f.* | *P* | *β* | *P* | *β* | *P* | *β* | *P* |
| Intercept | -0.919 | -0.641 | 133 | 0.523 | -1.091 | 0.419 | -4.264 | **0.031** | -8.654 | **0.044** |
| Emergence **time** (month) | 0.131 | 0.415 | 131 | 0.679 | 0.201 | 0.506 | 1.009 | **0.035** | 1.660 | **0.060** |
| Body **mass** (g) | 0.021 | 0.443 | 131 | 0.658 | 0.030 | 0.512 | 0.321 | **<0.001** | 0.292 | **0.033** |
| Nest:Cavity | -2.954 | -1.265 | 130 | 0.208 |  |  |  |  |  |  |
| Nest:Ground | -7.209 | -1.486 | 130 | 0.140 |  |  |  |  |  |  |
| Time × Mass | -0.007 | -0.593 | 131 | 0.553 | -0.009 | 0.405 | -0.080 | **<0.001** | -0.0656 | **0.0294** |
| Time × Nest:Cavity | 0.687 | 1.248 | 130 | 0.214 |  |  |  |  |  |  |
| Time × Nest:Ground | 1.440 | 1.402 | 130 | 0.163 |  |  |  |  |  |  |
| Mass × Nest:Cavity | 0.269 | 2.792 | 131 | **0.006** |  |  |  |  |  |  |
| Mass × Nest:Ground | 0.247 | 1.554 | 130 | 0.123 |  |  |  |  |  |  |
| Time × Mass × Nest:Cavity | -0.064 | -2.596 | 131 | **0.011** |  |  |  |  |  |  |
| Time × Mass × Nest:Ground | -0.055 | -1.577 | 129 | 0.117 |  |  |  |  |  |  |
| **3-way interaction** | **χ^2^=** | **8.688** | **2** | **0.013** |  |  |  |  |  |  |

Table S2: The results of fitted models for European birds

| **Variable** | **Full model (*n*=73)** | | | | **Branch (*n*=29)** | | **Cavity (*n*=25)** | | **Ground (*n*=19)** | |
| --- | --- | --- | --- | --- | --- | --- | --- | --- | --- | --- |
|  | *β* | *t* | *d.f.* | *P* | *β* | *P* | *β* | *P* | *β* | *P* |
| Intercept | -0.739 | -1.811 | 61 | 0.075 | -0.791 | 0.063 | -0.216 | 0.392 | -0.135 | 0.855 |
| Emergence **time** (month) | 0.178 | 1.600 | 61 | 0.115 | 0.191 | 0.096 | 0.023 | 0.718 | 0.002 | 0.989 |
| Body **mass** (g) | 0.057 | 2.695 | 61 | **0.009** | 0.059 | **0.010** | 0.011 | **0.077** | -0.010 | 0.653 |
| Nest:Cavity | 0.586 | 1.236 | 61 | 0.221 |  |  |  |  |  |  |
| Nest:Ground | 0.988 | 1.180 | 61 | 0.242 |  |  |  |  |  |  |
| Time × Mass | -0.015 | -2.535 | 61 | **0.014** | -0.015 | **0.014** | -0.002 | 0.141 | 0.002 | 0.676 |
| Time × Nest:Cavity | -0.016 | -1.247 | 61 | 0.217 |  |  |  |  |  |  |
| Time × Nest:Ground | -0.267 | -1.387 | 61 | 0.171 |  |  |  |  |  |  |
| Mass × Nest:Cavity | -0.048 | -2.172 | 61 | **0.034** |  |  |  |  |  |  |
| Mass × Nest:Ground | -0.077 | -2.529 | 61 | **0.014** |  |  |  |  |  |  |
| Time × Mass × Nest:Cavity | 0.013 | 2.121 | 61 | **0.038** |  |  |  |  |  |  |
| Time × Mass × Nest:Ground | 0.019 | 2.561 | 61 | **0.013** |  |  |  |  |  |  |
| **3-way interaction** | **χ^2^=** | **7.626** |  | **0.022** |  |  |  |  |  |  |
