# Supplementary material for: Conflict over non-partitioned resources may explain between-species differences in declines: the anthropogenic competition hypothesis
Source: Behav Ecol Sociobiol. 2017 Jun 10;71(7):99. doi: 10.1007/s00265-017-2327-z (PMC5486810; doi:10.1007/s00265-017-2327-z)
Supplement: ESM 2 — (DOC 139 kb) [file 265_2017_2327_MOESM2_ESM.doc]

**SUPPLEMENTARY INFORMATION: MODEL IMPLEMENTATION**

for

Conflict Over Non-partitioned Resources May Explain

Between-Species Differences in Declines:

The Anthropogenic Competition Hypothesis

in

Behavioral Ecology and Sociobiology

by

**Andrew D. Higginson**

Centre for Research in Animal Behaviour, College of Life and Environmental Sciences,

University of Exeter a.higginson@exeter.ac.uk

Here, I provide a complete technical description of the model implementation. At a decision epoch *t* an individual is size *s* (Big *s=B* or Small *s=S*), has status *x* (Searcher *x=U*, Intruder *x=I* or Resident *x=R*), is in nest quality *q* (Poor *q=P* or Good *q=G*), and is opposed by opponent *p* (None *p=N*, Big *p=B* or Small *p=S*). At each decision epoch, the animal faces a binary choice: when it is floating (*x=U*) it chooses whether to rest (δ=0) or actively search (δ=1); when it is at a nest site ($x\in\{I,R\}$) it chooses whether to leave (δ=0) or make a claim (δ=1). In practice, this binary choice is modelled as a probability of choosing one action or the other as doing so aids convergence. The strategy is therefore a five-dimensional array with all the combinations of all variables for each iteration *i* of the procedure *i*: δ*_i_*(*s,x,q,p,t*). It is necessary to assume an initial distribution of individuals over the time period, ρ, where ρ*_i_*(*s,x,q,t*) is the number of individuals of each size, status, and in each nest quality at each time step in iteration *i*. The numbers of residents, intruders, and searchers respectively were initially set to evenly decreasing or increasing over time:

$\rho_{1}(s,R,q,T)=\frac{Q_{q}}{\sum Q}N_{S}\left(1-e^{-\frac{t}{T}}\right)$ (A1)

$$\rho_{1}(s,I,q,T)=\frac{Q_{q}}{\sum Q}N_{S}\frac{1}{5}\left(e^{-\frac{t}{T}}\right)$$

$$\rho_{1}(s,U,q,T)=N_{S}\frac{4}{5}\left(e^{-\frac{t}{T}}\right)$$

I initialize the value of the individual’s life *V* at *T* as

$V(s,x,q,p,T)=\left\{\begin{matrix} W_{q}\text{ if }x=R \\ 0\text{ if }x=I\text{ or }U \end{matrix}\right.$ (A2)

i.e. there is a reproductive pay-off *W_q_* that depends on nest quality *q* if the individual is a resident at the end of the time period. Body size *s* and opponent size *p* are irrelevant at time *T*.

For all *t<T* we must find the evolutionarily stable strategy δ*_i_*(*s,x,q,p,t*) that maximizes the reproductive pay-off, given the behavior of other individuals. In the following I denote δ*_i_*(*s,x,q,p,t*) by δ*_i_* for clarity. First consider the case where the individual is searching (*x=S*). If the decision between time *t* and *t* + 1 is δ*_ι_ *then the probability of finding a nest site is $\frac{\delta_{t}(N_{P}+N_{G})}{M}$ and the probability of being killed by a predator is δ*_ι_*μ. Note that the option of δ=0 allows individuals to delay emergence. A searcher becomes an intruder in a nest of quality *q* with resident of size *s* with probability

$P_{S\to I}=\delta_{i}(1-\mu)\frac{N_{q}}{M}$ (A3)

and becomes an intruder in an empty nest of quality *q* with probability

$P_{S\to I}=\delta_{i}(1-\mu)\frac{N_{q}-\sum_{s\in\{S,B\}} \rho_{i}(s,R,q,t)}{M}$ (A4)

In the nest, there is a resident of size *p* with probability

$\frac{\rho_{i}(s,R,q,t)}{N_{q}}$ (A5)

and the nest is empty with probability

$\frac{N_{q}-\sum_{s\in\{S,B\}} \rho_{i}(s,R,q,t)}{N_{q}}$ (A6)

The searcher stays as a searcher with probability

$P_{S\to S}=(1-\delta_{i})+\delta_{i}(1-\mu_{S})\left(1-\frac{N_{P}+N_{G}}{M}\right)$ (A7)

Next, I consider the case where an individual is an intruder. An intruder will become a searcher only if they do not stake a claim (since all fights are to the death), which happens with probability

$P_{I\to S}=(1-\delta_{i})$ (A8)

An intruder will become a resident if they make a claim and either the opponent retreats or the intruder wins a fight, which happens with probability

$P_{I\to R}=\delta_{i}\left[(1-\delta_{i-1}(p,R,q,s,t)+\delta_{i-1}(p,R,q,s,t)\cdot\gamma(s,I,q,p,t)\right]$ (A9)

where *I* indicates that the focal individual is an intruder and the opponent must be a resident.

An resident will become a searcher only if an intruder makes a claim and the resident retreats, which happens with probability

$P_{R\to S}=(1-\delta_{i})\delta_{i-1}(p,I,q,s,t)$ (A10)

An resident will remain a resident if the opponent retreats or the resident wins a fight and does not die from extrinsic mortality, which happens with probability

$P_{R\to R}=(1-\mu_{R})\left\langle1-\delta_{i-1}(p,I,q,s,t)+\delta_{i}[\delta_{i-1}(p,I,q,s,t)\cdot\gamma(s,R,q,p,t)]\right\rangle$ (A11)

where *R* indicates that focal individual is a resident, and the opponent must be an intruder.

A successfully claiming intruder or resident who is the resident of a nest of quality *q* in the next time step will be confronted in that next time step by an opponent of size *p* with probability

$\frac{\rho_{i}(s,I,q,t+1)}{M}$ (A12)

It has no intruder to the nest with probability

$1-\sum_{s\in\{S,B\}} \rho_{i}(s,I,q,t+1)$ (A13)

Given these ingredients (A3-A13), the dynamic operators are:

$\begin{matrix} V_{i+1}(s,U,q,p,t)=P_{S\to S}V_{i+1}(s,U,1,1,t+1)+ \\ P_{S\to I}\left[V_{i+1}(s,I,q,p,t+1)\frac{\rho_{i}(s,R,q,t)}{N_{q}}+V_{i+1}(s,I,q,p,t+1)\frac{N_{q}-\sum_{s\in\{S,B\}} \rho_{i}(s,R,q,t)}{N_{q}}\right] \end{matrix}$(A14)

for searchers,

$\begin{matrix} V_{i+1}(s,I,q,p,t)=P_{I\to S}V_{i+1}(s,U,q,p,t+1) \\ +P_{I\to R}\left[V_{i+1}(s,R,q,p,t+1)\frac{\rho_{i}(s,I,q,t+1)}{M}+V_{i+1}(s,R,q,p,t+1)\left(1-\sum_{s\in\{S,B\}} \rho_{i}(s,I,q,t+1)\right)\right] \end{matrix}$(A15)

for intruders, and

$\begin{matrix} V_{i+1}(s,R,q,p,t)=P_{R\to S}V_{i+1}(s,U,q,p,t+1) \\ +P_{R\to R}\left[V_{i+1}(s,R,q,p,t+1)\frac{\rho_{i}(s,I,q,t+1)}{M}+V_{i+1}(s,R,q,p,t+1)\left(1-\sum_{s\in\{S,B\}} \rho_{i}(s,I,q,t+1)\right)\right] \end{matrix}$(A16)

for residents.

Using the dynamic operators (A14–A16), the optimal decision for all states can be found by backwards recursion for the *Early* condition. This strategy was then run forward in Markov chains to calculate the resulting distribution of states ρ*_i+1_*(*s,x,q,t*). The backwards recursion process then found the best response of a single mutant to ρ*_i+1_*(*s,x,q,t*), and this process was repeated until the fitness array (A14-16) converged. Convergence was judged to have occurred when $\left|V_{i+1}-V_{i}\right|<10^{-6}$, which typically happened within 100 iterations. In order to smooth the fitness landscape and aid convergence, I assume errors in decision-making occur with a probability proportional to the canonical cost (error magnitude = 0.02, (McNamara *et al.*, 1997)) and that when the fitness consequences of the two options are equal, the option to search (if *x=U*) or fight (if *x*=*R* or *I*) is chosen. The number of available *Poor* and *Good* nest sites at the start of the *Late* period is given by

$N_{L,P}=N_{E,P}-\sum_{s\in\{S,B\}} \rho_{i+1}(s,R,P,T)$ (A17)

$$N_{L,G}=N_{E,G}-\sum_{s\in\{S,B\}} \rho_{i+1}(s,R,G,T)$$

The strategy in the *Late* period is then found in the same way as described above, given the reduced nest availability.

This entire process is then iterated over many years, with the initial number of individuals in the next year being a decelerating function of reproductive success and limited by the carrying capacity for each type (*K*):

$N_{s,y+1}=\frac{K\sum_{q\in(P,G\}} \rho_{i+1}(s,R,q,T)\times W_{q}}{K+\sum_{q\in(P,G\}} \rho_{i+1}(s,R,q,T)\times W_{q}}$ (A18)

The inclusion of the carrying capacity implies that the abundance of a particular species is self-limiting due to competition later in the colony cycle, because of competition for resources within species. I iterate, with the ESS response found for each year, until no value of *N_s,y+1_* changes by more than 0.01. This gives the stable mixture of species types in the environment. To assess the impact of reducing nest-site density I run forward Markov chains using values of *Q_q_* reduced by the proportion *d_q_* that declines gradually and at an even rate over 60 years. I assume that because of the short timescale the evolved strategy does not change, and on the basis of this can calculate the trajectory of abundance of each species type during the decline in nest availability.
