# Supplementary material for: Conflict over non-partitioned resources may explain between-species differences in declines: the anthropogenic competition hypothesis
Source: Behav Ecol Sociobiol. 2017 Jun 10;71(7):99. doi: 10.1007/s00265-017-2327-z (PMC5486810; doi:10.1007/s00265-017-2327-z)
Supplement: ESM 3 — (DOC 99 kb) [file 265_2017_2327_MOESM3_ESM.doc]

**SUPPLEMENTARY INFORMATION: STATISTICAL RESULTS**

for

Conflict Over Non-partitioned Resources May Explain

Between-Species Differences in Declines:

The Anthropogenic Competition Hypothesis

in

Behavioral Ecology and Sociobiology

by

**Andrew D. Higginson**

Centre for Research in Animal Behaviour, College of Life and Environmental Sciences,

University of Exeter a.higginson@exeter.ac.uk

Table S1: The results of fitted models for North American birds. Dependent variable is the trend since 1966. *P* values for the effect of time, mass and their interaction were directed (0.625 × two-tailed *P*).

| **Variable** | **Full model (*n*=147)** | | | | **Branch (*n*=79)** | | **Cavity (*n*=38)** | | **Ground (*n*=30)** | |
| --- | --- | --- | --- | --- | --- | --- | --- | --- | --- | --- |
|  | *β* | *t* | *d.f.* | *P* | *β* | *P* | *β* | *P* | *β* | *P* |
| Intercept | -3.874 | -2.015 | 135.0 | 0.046 | -1.091 | 0.419 | -4.264 | 0.031 | -8.654 | 0.044 |
| Emergence time (month) | 0.818 | 1.788 | 131.9 | 0.048 | 0.201 | 0.316 | **1.009** | **0.022** | 1.660 | 0.037 |
| **Body mass (g)** | **0.290** | **3.430** | **132.4** | **<0.001** | 0.030 | 0.320 | **0.321** | **<0.001** | **0.292** | **0.021** |
| **Time × Mass** | **-0.070** | **-3.217** | **132.1** | **0.001** | -0.009 | 0.253 | **-0.080** | **<0.001** | **-0.066** | **0.016** |
| Nest:Ground | -4.256 | -0.856 | 129.8 | 0.393 |  |  |  |  |  |  |
| Nest:Branch | 2.954 | 1.265 | 130.4 | 0.208 |  |  |  |  |  |  |
| Time × Nest: Ground | 0.753 | 0.703 | 129.7 | 0.483 |  |  |  |  |  |  |
| Time × Nest: Branch | -0.687 | -1.248 | 130.2 | 0.214 |  |  |  |  |  |  |
| Mass × Nest: Ground | -0.022 | -0.127 | 129.6 | 0.899 |  |  |  |  |  |  |
| **Mass × Nest: Branch** | **-0.269** | **-2.792** | **131.2** | **0.006** |  |  |  |  |  |  |
| Time × Mass × Nest: Ground | 0.009 | 0.224 | 129.8 | 0.823 |  |  |  |  |  |  |
| **Time × Mass × Nest: Branch** | **0.064** | **2.596** | **131.0** | **0.010** |  |  |  |  |  |  |
| **3-way interaction** | **χ^2^=** | **8.688** | **2** | **0.013** |  |  |  |  |  |  |

Table S2: The results of fitted models for European birds. Dependent variable is the cube root (to normalize) of the trend since 1980. *P* values for the effect of time, mass and their interaction were directed (0.625 × two-tailed *P*).

| **Variable** | **Full model (*n*=73)** | | | | **Branch (*n*=29)** | | **Cavity (*n*=25)** | | **Ground (*n*=19)** | |
| --- | --- | --- | --- | --- | --- | --- | --- | --- | --- | --- |
|  | *β* | *t* | *d.f.* | *P* | *β* | *P* | *β* | *P* | *β* | *P* |
| Intercept | -0.739 | -1.811 | 60.5 | 0.075 | -0.791 | 0.063 | -0.216 | 0.392 | -0.135 | 0.855 |
| Emergence time (month) | 0.178 | 1.600 | 61.0 | 0.072 | 0.191 | 0.060 | 0.023 | 0.449 | 0.002 | 0.618 |
| Body mass (g) | **0.057** | **2.695** | **60.4** | **0.006** | **0.059** | **0.006** | **0.011** | **0.048** | -0.010 | 0.408 |
| Time × Mass | **-0.015** | **-2.535** | **60.5** | **0.008** | **-0.015** | **0.009** | -0.002 | 0.088 | 0.002 | 0.421 |
| Nest:Cavity | 0.586 | 1.236 | 61.0 | 0.221 |  |  |  |  |  |  |
| Nest:Ground | 0.988 | 1.180 | 60.3 | 0.242 |  |  |  |  |  |  |
| Time × Nest:Cavity | -0.016 | -1.247 | 61.0 | 0.217 |  |  |  |  |  |  |
| Time × Nest:Ground | -0.267 | -1.387 | 60.0 | 0.171 |  |  |  |  |  |  |
| Mass × Nest:Cavity | **-0.048** | **-2.172** | **60.6** | **0.034** |  |  |  |  |  |  |
| Mass × Nest:Ground | **-0.077** | **-2.529** | **61.0** | **0.014** |  |  |  |  |  |  |
| Time × Mass × Nest:Cavity | **0.013** | **2.121** | **60.6** | **0.038** |  |  |  |  |  |  |
| Time × Mass × Nest:Ground | **0.019** | **2.561** | **61.0** | **0.013** |  |  |  |  |  |  |
| **3-way interaction** | **χ^2^=** | **7.626** |  | **0.022** |  |  |  |  |  |  |

Table S3: The results of fitted models for worldwide bumblebees. Dependent variable is the arcsine of the proportional declines, over various periods (see original data sets). . *P* values for the effect of time, mass and their interaction were directed (0.625 × two-tailed *P*). Nest type was classified as surface (always or sometimes surface nesting) or underground (always underground nesting)

| **Variable** | **No nest type (*n*=61)** | | | | **No Chinese fauna (*n*=48)** | | | | **Chinese fauna assigned nest type (*n*=61)** | | | | | |
| --- | --- | --- | --- | --- | --- | --- | --- | --- | --- | --- | --- | --- | --- | --- |
|  |  |  |  |  |  |  |  |  | Surface | | Underground | | Random | |
|  | *β* | *t* | *d.f.* | *P* | *β* | *t* | *d.f.* | *P* | *β* | *P* | *β* | *P* | *β* | *P* |
| Intercept | -0.818 | -2.008 | 4.0 | 0.114 | -0.745 | -1.661 | 3.36 | 0.186 | -0.607 | 0.187 | -0.528 | 0.285 | -0.635 | 0.177 |
| Relative body mass | -0.042 | -1.150 | 41.6 | 0.160 | -0.093 | -0.946 | 6 | 0.220 | -0.006 | 0.539 | -0.008 | 0.822 | -0.016 | 0.658 |
| Relative emergence time | **-0.211** | **-3.818** | **47.0** | **<0.001** | **-0.295** | **-3.660** | **33.5** | **<0.001** | **-0.224** | **<0.001** | **-0.224** | **<0.001** | **-0.224** | **<0.001** |
| Nest type |  |  |  |  | **-0.500** | **-3.046** | **35.7** | **0.004** | **-0.462** | **0.003** | **-0.442** | **0.002** | **-0.387** | **0.005** |
| Mass × Time | **-0.052** | **-1.970** | **52.4** | **0.034** | -0.114 | -1.599 | 31.6 | 0.075 | **-0.055** | **0.018** | **-0.055** | **0.018** | **-0.054** | **0.023** |
| Mass × Nest type |  |  |  |  | 0.120 | 0.970 | 30.8 | 0.340 |  |  |  |  |  |  |
| Time × Nest type |  |  |  |  | 0.136 | 1.120 | 37.4 | 0.270 |  |  |  |  |  |  |
| Mass × Time × Nest type |  |  |  |  | 0.045 | 0.562 | 35.8 | 0.578 |  |  |  |  |  |  |
