# Supplementary material for: Conflict over non-partitioned resources may explain between-species differences in declines: the anthropogenic competition hypothesis
Source: Behav Ecol Sociobiol. 2017 Jun 10;71(7):99. doi: 10.1007/s00265-017-2327-z (PMC5486810; doi:10.1007/s00265-017-2327-z)
Supplement: ESM 4 — (DOC 144 kb) [file 265_2017_2327_MOESM4_ESM.doc]

**SUPPLEMENTARY INFORMATION: SENSITIVITY ANALYSIS**

for

Conflict Over Non-partitioned Resources May Explain

Between-Species Differences in Declines:

The Anthropogenic Competition Hypothesis

in

Behavioral Ecology and Sociobiology

by

**Andrew D. Higginson**

Centre for Research in Animal Behaviour, College of Life and Environmental Sciences,

University of Exeter a.higginson@exeter.ac.uk

**Sensitivity Analysis**

Here, I explore the dependence of the cross-over interaction predicted in Fig. 2B on the assumed values of the critical parameters. The results of this sensitivity analysis are shown in Fig. S1. The size advantage in contests *α* has no impact on the relative declines of the four species types (Fig. S2A), whilst the resident advantage *β* has no effect unless it is much larger than *α* (Fig. S2B, and true for all *α*) because it no longer pays *Late Big* species to hold out for the best sites. The relative declines of the four types is influenced by the payoff from *Poor* sites (Fig. S2C), with *Late Big* types declining more than others only if the payoff is very different to the payoff from *Good* sites (unless the payoff is zero: i.e. there are no *Poor* sites). When all sites give similar payoffs *Big* types tend to decline less than *Small* types because *Late Big* individuals claim all nest types and so competitive ability becomes more important. The number of places to search *M,* which controls the probability of finding a nest site per time step, has no effect on the relative declines (Fig. S2D), although as *M* increases and sites become more difficult to find the effects of size and timing get smaller. The proportion of sites that have been lost that were *Good* has stronger effects on the particular pattern that we observe (Fig. S2E). When *Poor* nest sites have been lost much more than *Good* sites the decline of *Late Small* types is predicted to be greater than the decline of *Late Big* types because *Late Small* types preferentially nest in *Poor* sites. If, on the other hand, most nest-site loss is of *Good* sites, *Early* types are predicted to decline more because they preferentially occupy *Good* sites. The interaction is not found when mortality whilst searching *μ_S_* is very high (Fig. S2F) because individuals should accept the first nest they find, nor if mortality is zero because *Late Big* individuals do not suffer increased mortality when searching for scarcer *Good* sites. Thus, the model predicts the cross-over interaction for almost all reasonable parameter settings.


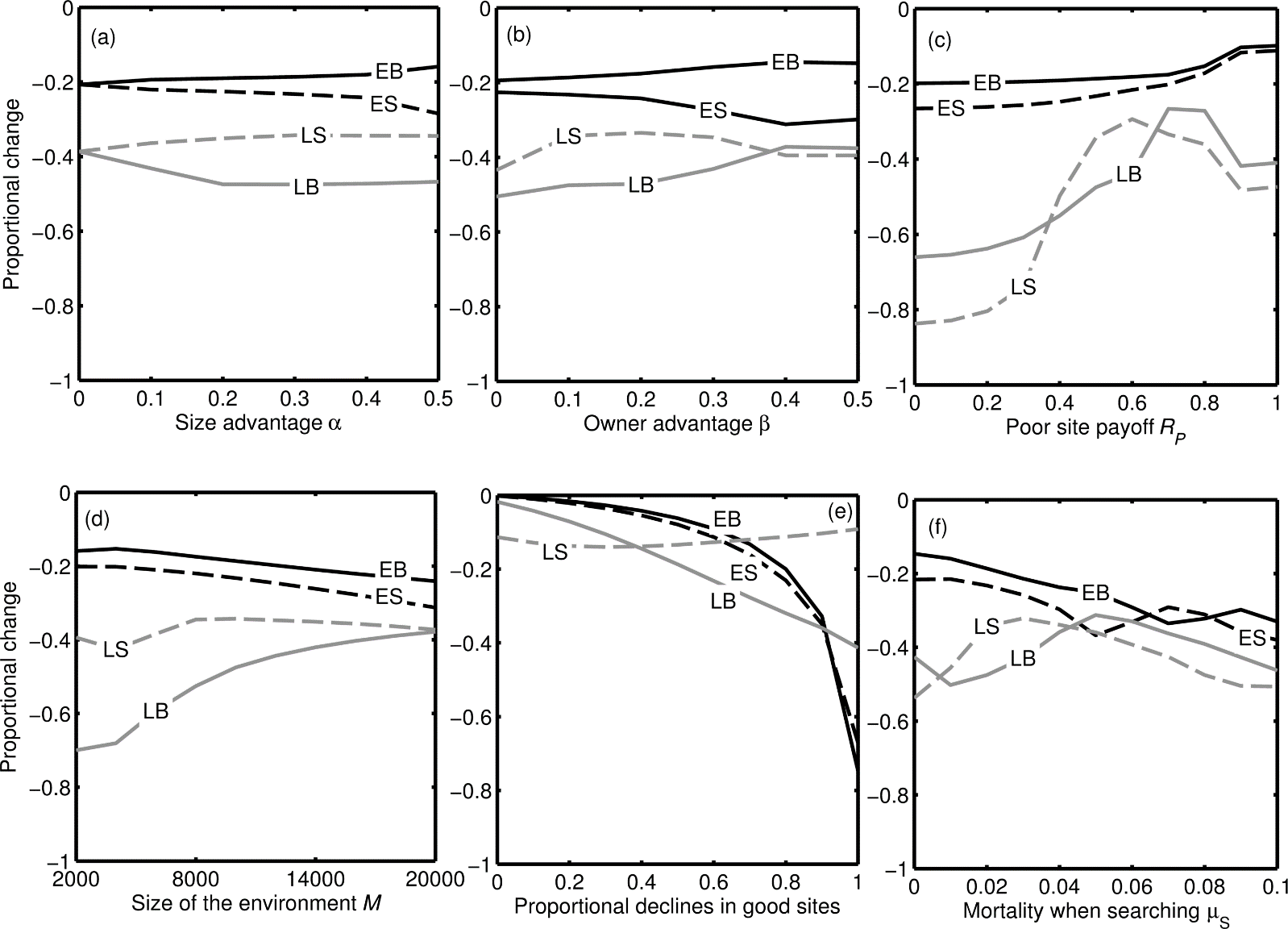


**Fig. S1:** Proportional change for the four types indicated by *Early* (*E*, black lines) or *Late* (*L*, grey lines) and *Small* (*S*, dashed lines) or *Big* (*B*, solid lines) as a function of (a) size advantage in fights *α*, (b) resident advantage in fights *β*, (c) payoff from poor nest sites *W_P_*, (d) number of places to search *M*, (e) proportional decline in the abundance of *Good* nest sites [*d_G_*/(*d_P_*+*d_G_*)], where *d_P_* + *d_G_* = 0.75, and (f) mortality rate when searching μ_S_.
